# Homer binds to Orai1 and TRPC channels in the neointima and regulates vascular smooth muscle cell migration and proliferation

**DOI:** 10.1038/s41598-017-04747-w

**Published:** 2017-07-11

**Authors:** Shuping Jia, Miguel Rodriguez, Arthur G. Williams, Joseph P. Yuan

**Affiliations:** Institute for Cardiovascular & Metabolic Diseases, University of North Texas Health Sciences Center, Fort Worth, TX 76107 USA

## Abstract

The molecular components of store-operated Ca^2+^ influx channels (SOCs) in proliferative and migratory vascular smooth muscle cells (VSMCs) are quite intricate with many channels contributing to SOCs. They include the Ca^2+^-selective Orai1 and members of the transient receptor potential canonical (TRPC) channels, which are activated by the endoplasmic reticulum Ca^2+^ sensor STIM1. The scaffolding protein Homer assembles SOC complexes, but its role in VSMCs is not well understood. Here, we asked whether these SOC components and Homer1 are present in the same complex in VSMCs and how Homer1 contributes to VSMC SOCs, proliferation, and migration leading to neointima formation. Homer1 expression levels are upregulated in balloon-injured vs. uninjured VSMCs. Coimmunoprecipitation assays revealed the presence and interaction of all SOC components in the injured VSMCs, where Homer1 interacts with Orai1 and various TRPC channels. Accordingly, knockdown of Homer1 in cultured VSMCs partially inhibited SOCs, VSMC migration, and VSMC proliferation. Neointimal area was reduced after treatment with an adeno-associated viral vector expressing a short hairpin RNA against Homer1 mRNA (AAV-shHomer1). These findings stress the role of multiple Ca^2+^ influx channels in VSMCs and are the first to show the role of Homer proteins in VSMCs and its importance in neointima formation.

## Introduction

Receptor-evoked Ca^2+^ signal consists of release of Ca^2+^ from the endoplasmic reticulum (ER)^1^ followed by activation of store-operated Ca^2+^ influx channels (SOCs). Components of SOCs include the Orai family of channels and the transient receptor potential subfamily C (TRPC) channels, and both channels are gated by the ER Ca^2+^ sensor STIM1^1–4^. STIM1 has an N-terminal, ER-resident EF hand that binds Ca^2+^ and a SAM domain that participates in clustering of STIM1 upon ER Ca^2+^ store depletion. The C-terminal, cytosolic portion of STIM1 consists of an inhibitory coiled-coil domain (CC1) that interacts with the V-shaped STIM1 Orai-activating region (SOAR)^5, 6^. SOAR is followed by the C-terminal inhibitory domain (CTID) that allows access of the STIM1 regulator SARAF to SOAR, a serine/proline region, and a polybasic, lysine-rich domain (K-domain)^7^.

Orai1 is a hexamer of four transmembrane-spanning units^8^ that functions as a highly Ca^2+^-selective, inward-rectifying channel and is the CRAC (Ca^2+^ release-activated Ca^2+^) pore-forming unit. Orai1 is activated by SOAR when STIM1 oligomerizes and accumulates at ER-PM junctions to bind to Orai1 after ER Ca^2+^ store depletion. The function and regulation of the TRPC channels by STIM1 have been documented in multiple cell types and for most TRPC channels. STIM1 interacts directly with TRPC1, TRPC4, and TRPC5, and indirectly with TRPC3 and TRPC6 by heteromultimerizing (a) TRPC1 with TRPC3 and (b) TRPC4 with TRPC6, allowing STIM1 to regulate TRPC3 and TRPC6^1^. STIM1 SOAR interacts with both the N- (NT) and C-terminal (CT) TRPC1 coiled-coil domains (CCDs). When TRPC1 is in complex with TRPC3, cell stimulation facilitates the interaction of SOAR with the CT CCDs of TRPC1 and TRPC3^9^. However, SOAR is not sufficient to activate TRPC channels, and gating of TRPC channels by STIM1 requires an additional electrostatic interaction of two conserved end lysines on STIM1 with two complementary aspartates/glutamates in the C-terminus of TRPC channels^4, 10^.

TRPC channels are also regulated by Homer proteins. We have previously shown that Homer binds to multiple family members of TRPC channels as well as to IP_3_ receptors (IP_3_Rs) to form a TRPC1/3-Homer-IP_3_R complex^11^. This complex stabilizes TRPC channels and prevents their spontaneous activation and Ca^2+^ influx in resting cells. Homer also regulates the translocation and retrieval of TRPC3 to and from the plasma membrane in response to ER Ca^2+^ store levels and IP_3_R activity^12^. In agonist-induced platelet aggregation, Homer is involved in STIM1-Orai1 interaction mediating SOC entry (SOCE)^13^. Homers are scaffolding proteins that, in addition to TRPC channels and IP_3_Rs, bind to several Ca^2+^-signaling proteins (ryanodine receptors, NFATc, Group I metabotropic glutamate receptors, Shank)^14, 15^. Homer1 was initially identified in neurons at the postsynaptic density to regulate dendrite morphogenesis and synaptic plasticity^16^. Homer1a, the founding isoform discovered as a neuronal immediate early gene, lacks the multimerizing coiled-coil domain and functions as a negative regulator of the long Homers^17^. Both Homers have EVH domains that bind to consensus proline-rich sequence (PXXF) present in target Ca^2+^-signaling proteins^18^.

Store-operated calcium entry (SOCE) is a major contributor of vascular smooth muscle cell (VSMC) phenotypic changes that lead to their proliferation and migration. In its normal state, VSMCs play a critical role in maintaining the integrity of the vessel wall and controlling vascular tone. VSMCs are normally quiescent, differentiated, and contractile. When the blood vessel wall is damaged from underlying factors, such as atherosclerosis, diabetes, and hypertension, VSMCs undergo pathological changes and dedifferentiate, proliferate, and migrate into the vessel lumen^19^. The subsequent extracellular matrix production and neointima formation result in narrowing of the lumen, leading to occlusive arterial disease. It is clear that STIM1 and Orai1 are critical components of SOCE in VSMC proliferation and migration, as revealed by their knockdown using siRNA^20, 21^. TRPC1 has also been shown to be a significant component of proliferative VSMC SOCs since application of either siTRPC1 or anti-TRPC1 antibody attenuates SOCE^22, 23^. In addition, TRPC3, TRPC4, and TRPC6 are also major components of Ca^2+^ influx in arterial smooth muscle cells^24^. Targeting individual Ca^2+^ influx channels for therapy may raise a problem due to redundancy and compensation. An alternative approach is to target a non-essential protein that is a common component of all channels, such as the scaffolding protein Homer1. However, how the Homer proteins function in SOCs and SOC-dependent activities in VSMCs is not known.

In the present work, we examine if Homer1 is required for VSMC SOCE, migration, and proliferation leading to neointima formation and stenosis. We report that expression of Homer1, along with STIM1, Orai1, and TRPC channels, is increased in the neointima after balloon injury in the rat carotid artery. Knockdown of Homer1 significantly reduces SOCE, VSMC migration and proliferation, and neointima size. These findings identify a key role for Homer1 in SOC function in injured VSMCs, resulting in occlusive arterial disease and suggest Homer1 as a preferred therapeutical target.

## Results

### Homer1 protein expression and interaction with TRPC channels and Orai1 are increased in neointimal VSMCs of balloon-injured rat carotid arteries

**Figure 1:**
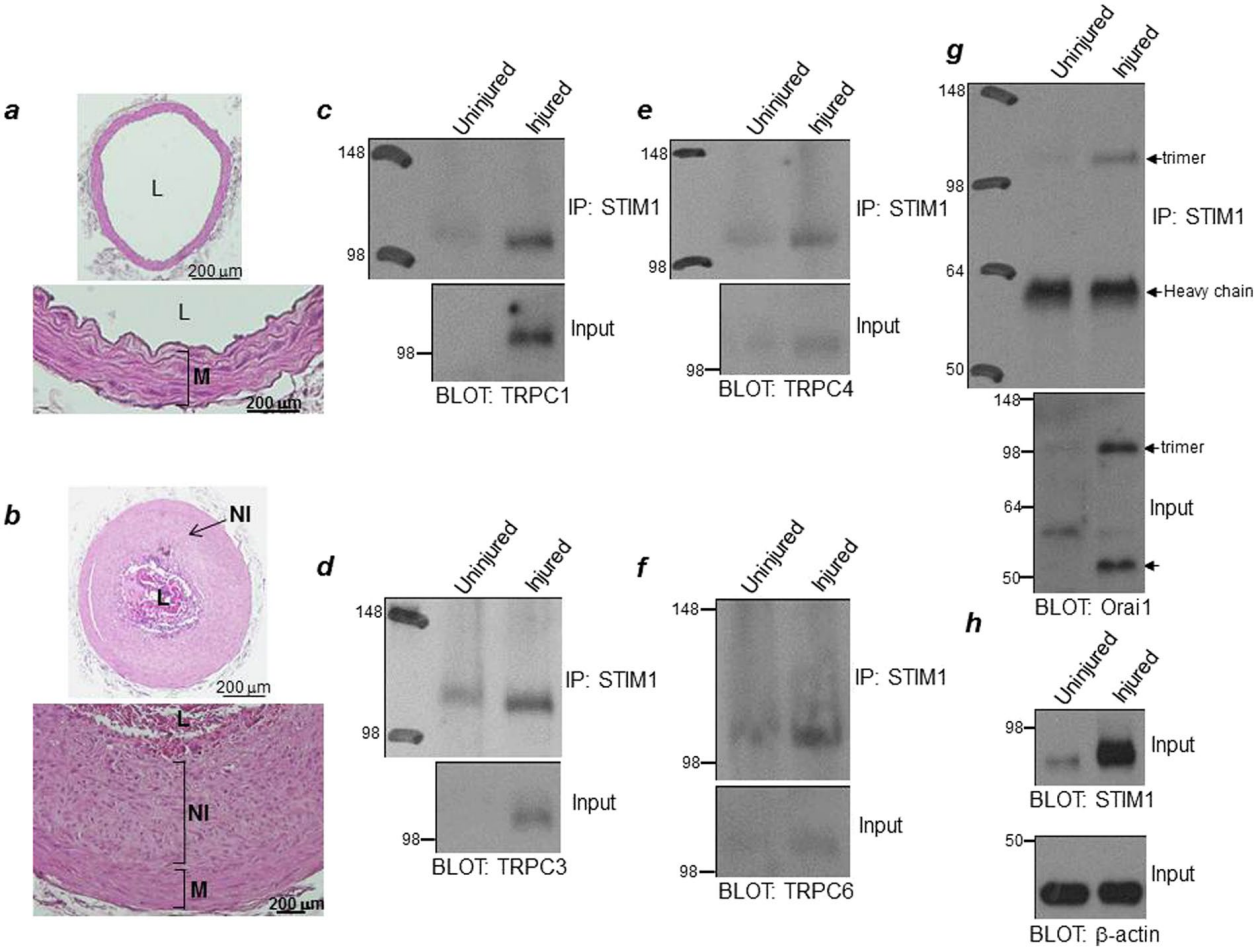
Increased protein expression and interaction of TRPC channels and Orai1 with STIM1 in neointima from balloon-injured rat carotid artery. H&E-stained sections of (**a**) sham-operated (intact) and (**b**) balloon-injured arteries 14 days after injury. Prominent neointima is present and narrows the lumen. Photos are taken at 10× and 40× magnification. M = media; NI = neointima; L = lumen. Protein expression levels of TRPC1, TRPC3, TRPC4, TRPC6, Orai1, and STIM1 (**c**–**h**) are upregulated in injured (media + neointima) compared to uninjured VSMCs. Co-immunoprecipitation assay using anti-STIM1 antibody to immunoprecipitate STIM1 shows increased interaction of STIM1 with (**c**) TRPC1; (**d**) TRPC3; (**e**) TRPC4; (**f**) TRPC6; and (**g**) Orai1 more strongly in injured VSMCs.

We performed balloon injury on left carotid arteries (CAs), using right carotid arteries as negative control (Fig. 1a). Neointima developed and peaked at 14 days postinjury (Fig. 1b), and the injured media and neointima from the CAs were isolated for protein expression and coimmunoprecipitation analyses. As reported by others^20, 21, 25, 26^, STIM1, Orai1, TRPC1, TRPC4, and TRPC6 expression levels are increased in injured medial and neointimal, proliferative VSMCs (collectively called ‘injured’) (Fig. 1c–h). We also analyzed TRPC3 expression level and found robust increase in TRPC3 in injured vs. uninjured VSMCs (Fig. 1d). Notably, an additional new and important finding is that the enhanced expression levels of these SOC components in injured VSMCs results in a greater number of SOC complexes to form and, likely, increased basal Ca^2+^ influx in comparison to that observed in uninjured VSMCs, as shown in the coimmunoprecipitation of STIM1 with Orai1 and TRPC channels (Fig. 1c–h).

**Figure 2:**
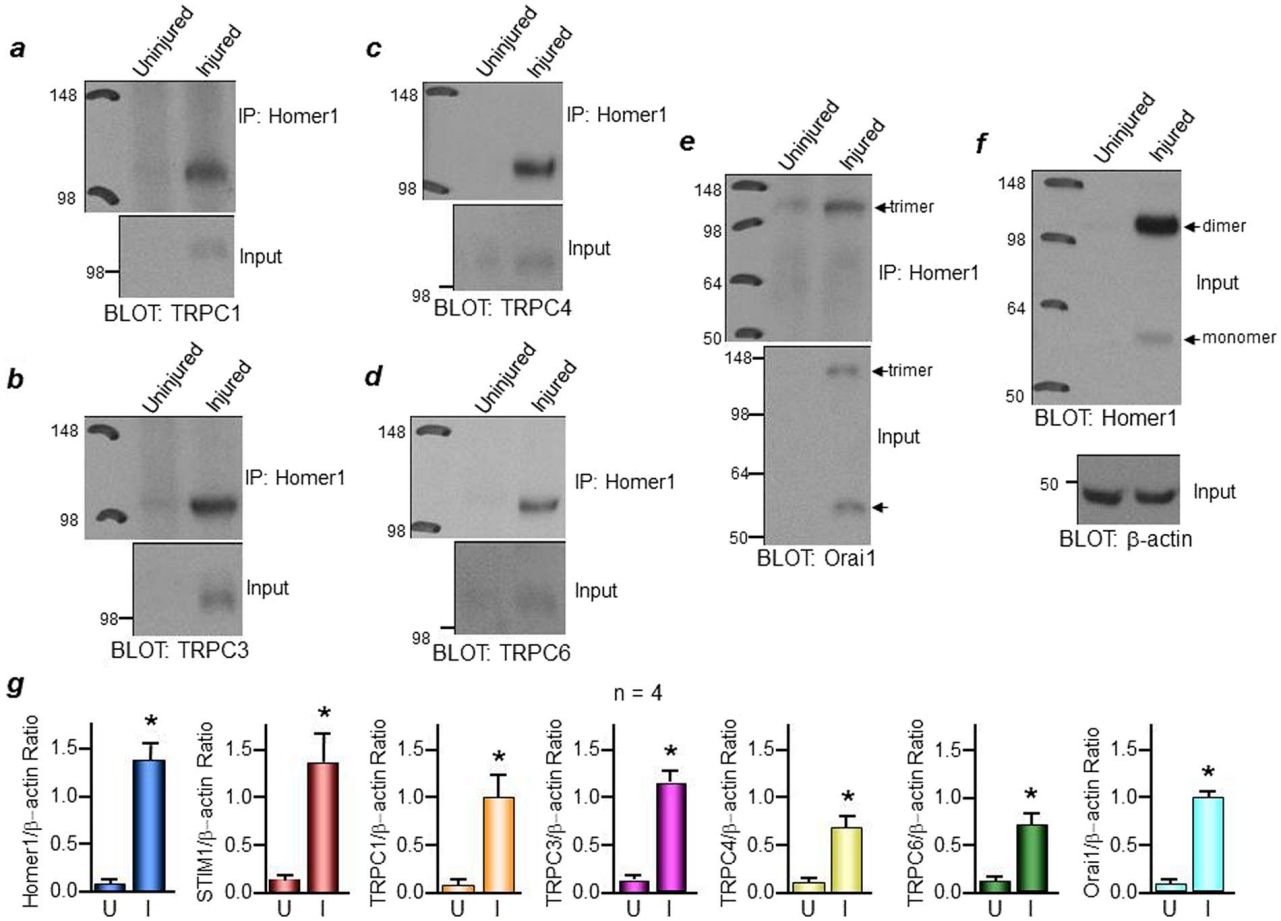
Increased protein expression and interaction of TRPC channels and Orai1 with Homer1 in neointima from balloon-injured rat carotid artery. Protein expression levels of Homer1 along with TRPC1, TRPC3, TRPC4, TRPC6, and Orai1 (**a**–**f**) are upregulated in injured (media + neointima) compared to uninjured VSMCs. Co-immunoprecipitation assay using anti-Homer1 antibody to immunoprecipitate Homer1 shows that Homer1 interacts with (**a**) TRPC1; (**b**) TRPC3; (**c**) TRPC4; (**d**) TRPC6; and (**e**) Orai1 in injured VSMCs. (**g**) Summary of protein expression normalized to β-actin in uninjured (U) and injured (I) VSMCs (n = 4). *p < 0.05 or better compared to uninjured VSMCs.

Increased basal and stimulated Ca^2+^ influx is toxic in many cells under different stressors, and it is expected that the cells will adapt to protect against the increased Ca^2+^ influx. An established native regulator of Ca^2+^ influx is Homer1^17^. Homer1 complexes TRPC channels to IP_3_ receptors to keep the channel in the closed state^11^, and interacts with TRPC channel C-terminal PXXF motif located next to the residues mediating gating of TRPC channels by STIM1^11^. Therefore, next, we examined if Homer1 expression levels increase, similar to STIM1, Orai1, and TRPC channels, and whether they interact with these proteins in the complexes. Homer1 protein was not detected in uninjured vessels, but its expression level markedly increases in injured VSMCs (Fig. 2f), along with STIM1, Orai1, TRPC1, TRPC3, TRPC4, and TRPC6. These increases in injured vs. uninjured arteries are quantified in Fig. 2g (n = 4). Co-immunoprecipitation with a Homer1 antibody shows that Homer1 interaction with Orai1 (Fig. 2e) as well as TRPC1, TRPC3, TRPC4, and TRPC6 increases due to their enhanced expression levels in injured arteries (Fig. 2a–d).

### Role of Homer1 in store-operated calcium entry (SOCE) in cultured VSMCs

**Figure 3:**
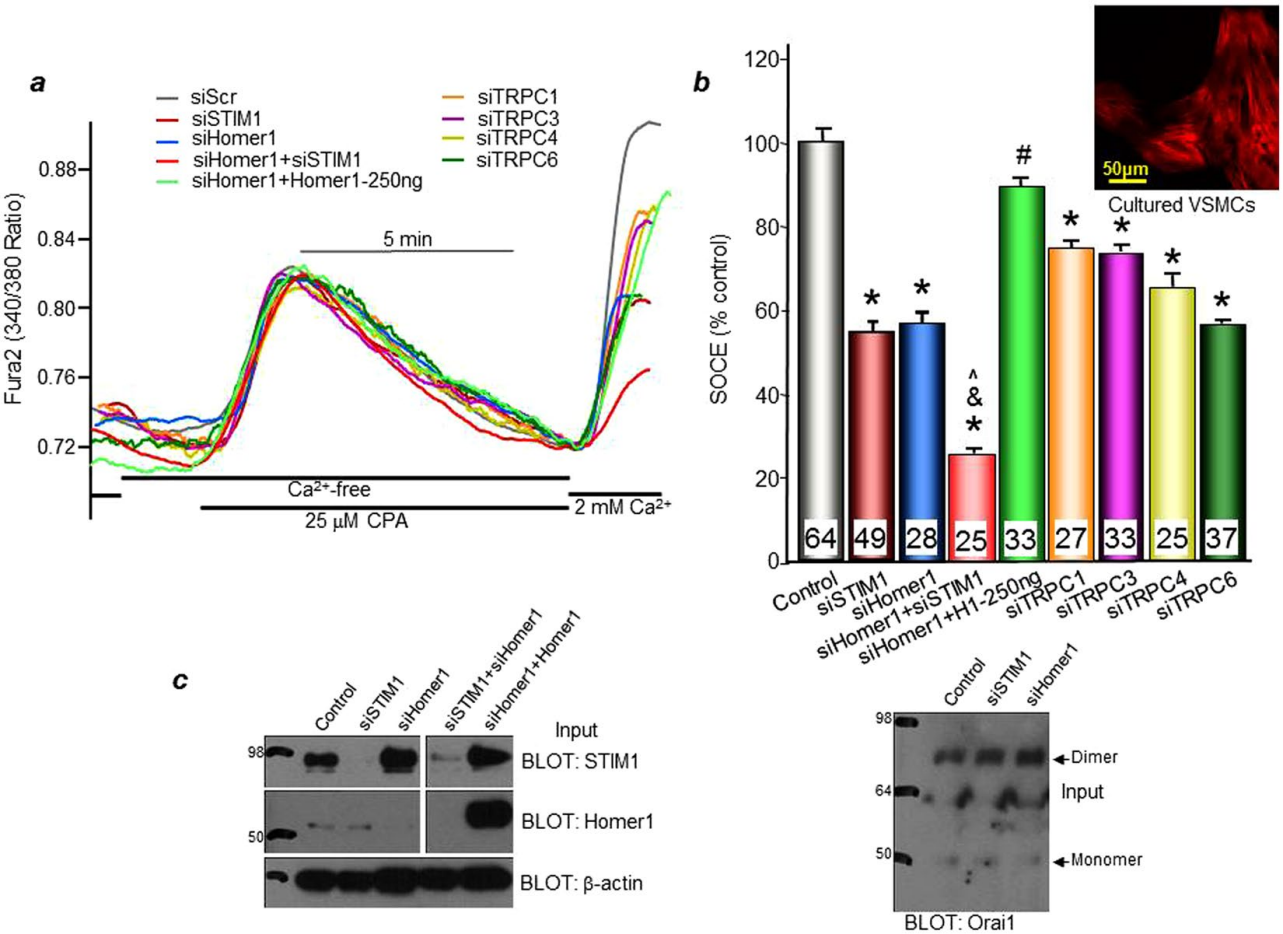
Effect of Homer1 knockdown on store-operated Ca^2+^ entry (SOCE) in cultured VSMCs. Cultured aortic VSMCs (1 × 10^5^) were transfected with various siRNAs and 250 ng/mL of Homer1 cDNA, as indicated, and SOCE was measured after depleting ER Ca^2+^ stores with 25 μM cyclopiazonic acid (CPA). (**a**) Representative Ca^2+^ traces vs. time. (**b**) SiHomer1, siSTIM1, siHomer1 + siSTIM1, siTRPC1, siTRPC3, siTRPC4, and siTRPC6 all significantly reduce SOCE in cultured VSMCs. Knockdown of both Homer1 and STIM1 further inhibits SOCE and, thus, has an additive effect on Ca^2+^ influx. By contrast, Homer1 overexpression rescues inhibition of SOCE by siHomer1. SOCE values are normalized to peak Ca^2+^ store depletion (CPA response) and indicated as % control (scrambled siRNA), which is set to 100%. *p < 0.01 compared to control; ^#^p < 0.01 compared to siHomer1; ^^^p < 0.01 compared to siSTIM1; ^&^p < 0.01 compared to siHomer1. Representative VSMCs stained with β-actin (red) are shown (inset). (**c**) Protein expression levels of native Homer1 and STIM1 are reduced in cells treated with their respective siRNAs, while Homer1 overexpression is robust with no effect of siHomer1. Spliced images come from the corresponding same blot, but are not neighboring lanes.

We investigated if upregulated Homer1, with its binding to known SOCs (i.e. TRPC channels and Orai1) in injured VSMCs, contributes to SOCE. Rat aortic VSMCs were isolated and cultured to allow phenotypic modulation to proliferative and migratory state that occurs during culturing and is similar to what happens in balloon-injured VSMCs. Hallmarks of this change include elevated SOCE leading to this increased rate of proliferation and migration^27, 28^. We used siRNA technology to target Homer1 and to determine how this knockdown affects SOCE and VSMC proliferation and migration in cultured VSMCs. SOCE was assessed by measuring Ca^2+^ entry (2 mM external) after store depletion using the SERCA pump inhibitor cyclopiazonic acid (CPA) in the absence of external Ca^2+^. Cells treated with siSTIM1 and/or siHomer1 have reduced STIM1/Homer1 mRNA (Supplemental Fig. 1) and protein (Fig. 3c) expression levels, respectively. Representative Ca^2+^ traces are shown in Fig. 3a. SOCE values are normalized to peak Ca^2+^ store depletion and are expressed as % control, which is set to 100%. Cells treated with siHomer1 exhibit significant reduction in SOCE (56.0 ± 4.0%) compared to control (scrambled siRNA), similar to a reduction in SOCE seen in cells treated with siSTIM1 (54.1 ± 2.8%) (Fig. 3b). Notably, the combination knockdown of STIM1 and Homer1 (siHomer1 + siSTIM1) has an additive, significant effect further inhibiting SOCE compared to their individual knockdowns, resulting in a residual SOCE of only 22.7 ± 2.0% and indicating that siHomer1 and siSTIM1 act independently of each other. The reduction of SOCE by siHomer1 can be rescued by overexpressing exogenous Homer1 resistant to siHomer treatment (siHomer1 + Homer1 (90.6 ± 3.1%)) (Fig. 3c). VSMCs treated with siTRPC1, siTRPC3, siTRPC4, or siTRPC6 also show significant suppression of SOCE with results of 78.2 ± 2.3%, 74.7 ± 2.4%, 65.0 ± 3.8%, or 55 ± 1.4% residual activity, respectively. Two independent siRNAs for Homer1 and TRPC1 showed similar effects on reducing SOCE in cultured VSMCs (Supplementary Fig. 2), with the two Homer1 siRNAs targeting the 3′UTR and the two TRPC1 siRNAs targeting the cDNA region. These observations indicate that both Homer1 and STIM1 as well as TRPC channels are essential for SOCE in proliferative and migratory VSMCs. Moreover, the additive effects of siSTIM1 and siHomer1 on the Ca^2+^ influx suggest that TRPC channels can form two types of channels in VSMC - STIM1-regulated and Homer1-regulated channels. Alternatively, knockdown of STIM1 may inhibit Ca^2+^ influx by Orai1 while knockdown of Homer1 may inhibits Ca^2+^ influx by TRPC channels. The coimmunoprecipitation of all these proteins may result from their presence in the same complexes and the same microdomain.

### Role of Homer1 in VSMC migration and proliferation

**Figure 4:**
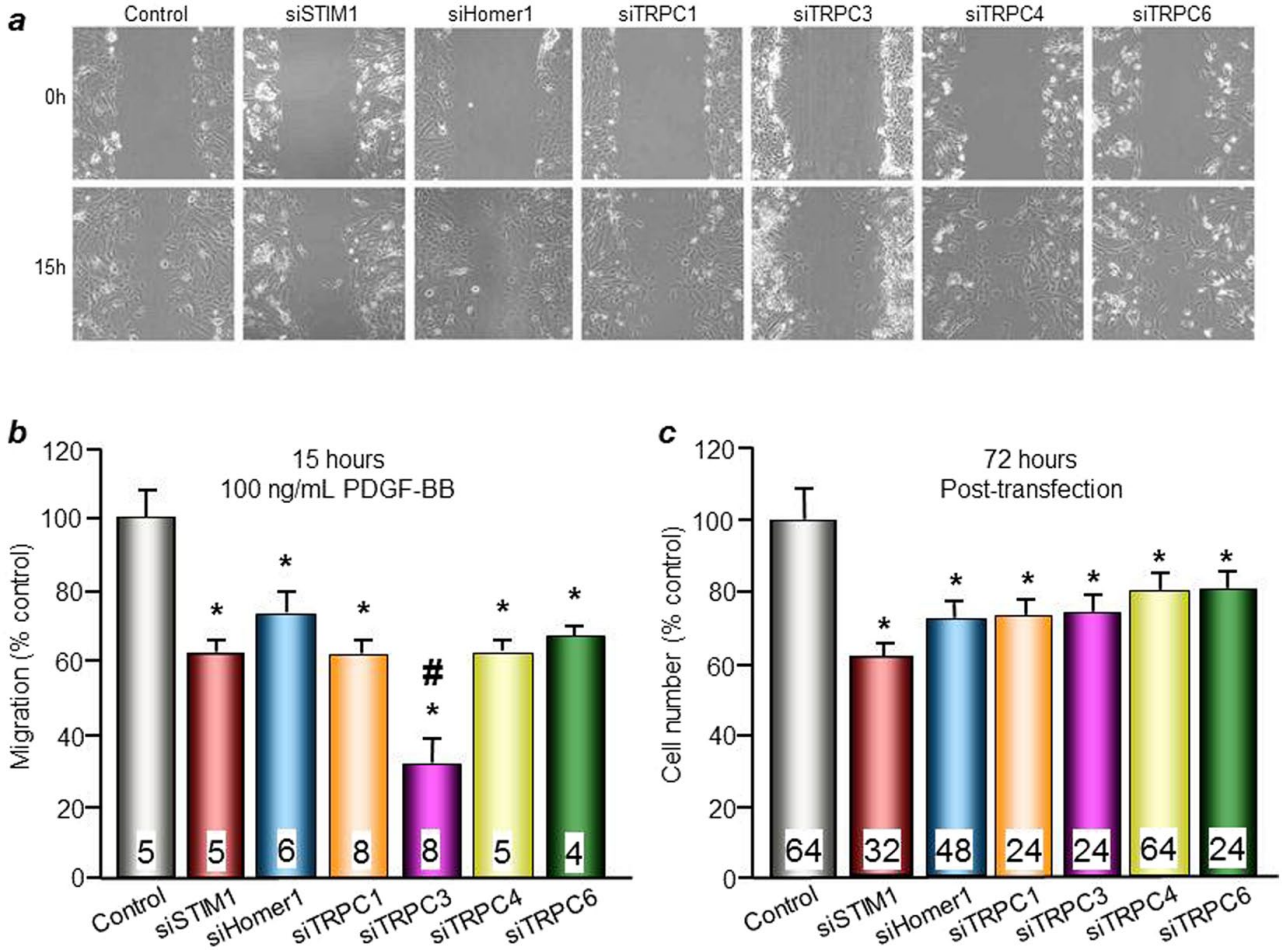
Effect of Homer1 knockdown on VSMC migration and proliferation. Cultured aortic VSMCs were transfected with various siRNAs, as indicated. (**a**) Representative brightfield views of scratch wound migration assay with 100 ng/mL PDGF-BB at 0 hr and 15 hrs and (**b**) Quantification of VSMC migration after 15 hrs as % control (% scrambled siRNA = 100%) compared to 0 hrs. SiHomer1, siSTIM1, siTRPC1, siTRPC3, siTRPC4, and siTRPC6 all reduce migration, with siTRPC3 being the lowest. *p < 0.05 or better compared to control ^#^p < 0.01 compared to other 5 treatment groups and excludes control. (**c**) Proliferation of VSMCs in complete media containing 10% serum, as % control (% scrambled siRNA = 100%). All conditions, except for control, decrease cell number. *p < 0.01 compared to control.

Next, we determined if Homer1 has any significant effects on VSMC migration. A scratch wound assay was done to determine VSMC migration in response to 100 ng/mL PDGF-BB. Cultured VSMCs were transfected with siHomer1, siSTIM1, siTRPC1, siTRPC3, siTRPC4, or siTRPC6 and incubated for 72 hrs, with the scratch wound made 15 hrs before analysis. Wound closures were analyzed postscratch to determine contribution from VSMC migration. Representative scratch wound analysis at 0 and 15 hrs postscratch are shown in Fig. 4a. Cells treated with siHomer1 migrate significantly less over the wound surface area (73.3 ± 5.9%) compared to control (scrambled siRNA) (Fig. 4b). Cells treated with siSTIM1 (62.1 ± 4.0%), siTRPC1 (63.4 ± 3.5%), siTRPC3 (32.3 ± 5.8%), siTRPC4 (62.4 ± 2.7%), or siTRPC6 (67.5 ± 2.2%) also show significant reductions in migration after 15 hours compared to control. In particular, cells treated with siTRPC3 have significantly lower migration compared to cells treated with siHomer1, siSTIM1, and the other siTRPCs, indicating that TRPC3 is a major Ca^2+^ influx channel regulating PDGF-BB-induced VSMC migration. It is well established that receptor complexes are formed with specific complement of signaling proteins^29^. Our findings suggest that TRPC3 is the main Ca^2+^ influx channel activated by the PDGF receptors in VSMCs^30^.

VSMC proliferation was assessed in response to 10% serum in complete media. Cultured VSMCs were transfected with siHomer1, siSTIM1, siTRPC1, siTRPC3, siTRPC4, or siTRPC6, and plated onto 96-well plate. MTT assay was performed, and absorbance readings of solubilized formazan, whose amount of insoluble form is directly proportional to the cell number, were taken at 570 nm. Cells treated with siHomer1 proliferate significantly less (73.3 ± 4.2%) compared to control (scrambled siRNA) (Fig. 4c). Significant reductions in proliferation were also seen in cells treated with siSTIM1 (62.7 ± 3.0%), siTRPC1 (73.5 ± 4.0%), siTRPC3 (74.1 ± 3.5%), siTRPC4 (79.5 ± 4.9%), or siTRPC6 (80.9 ± 3.9%). Hence, our results indicate that Homer1 is necessary for VSMC migration and proliferation.

### *In vivo* infection with shHomer1 adeno-associated virus reduces neointima

**Figure 5:**
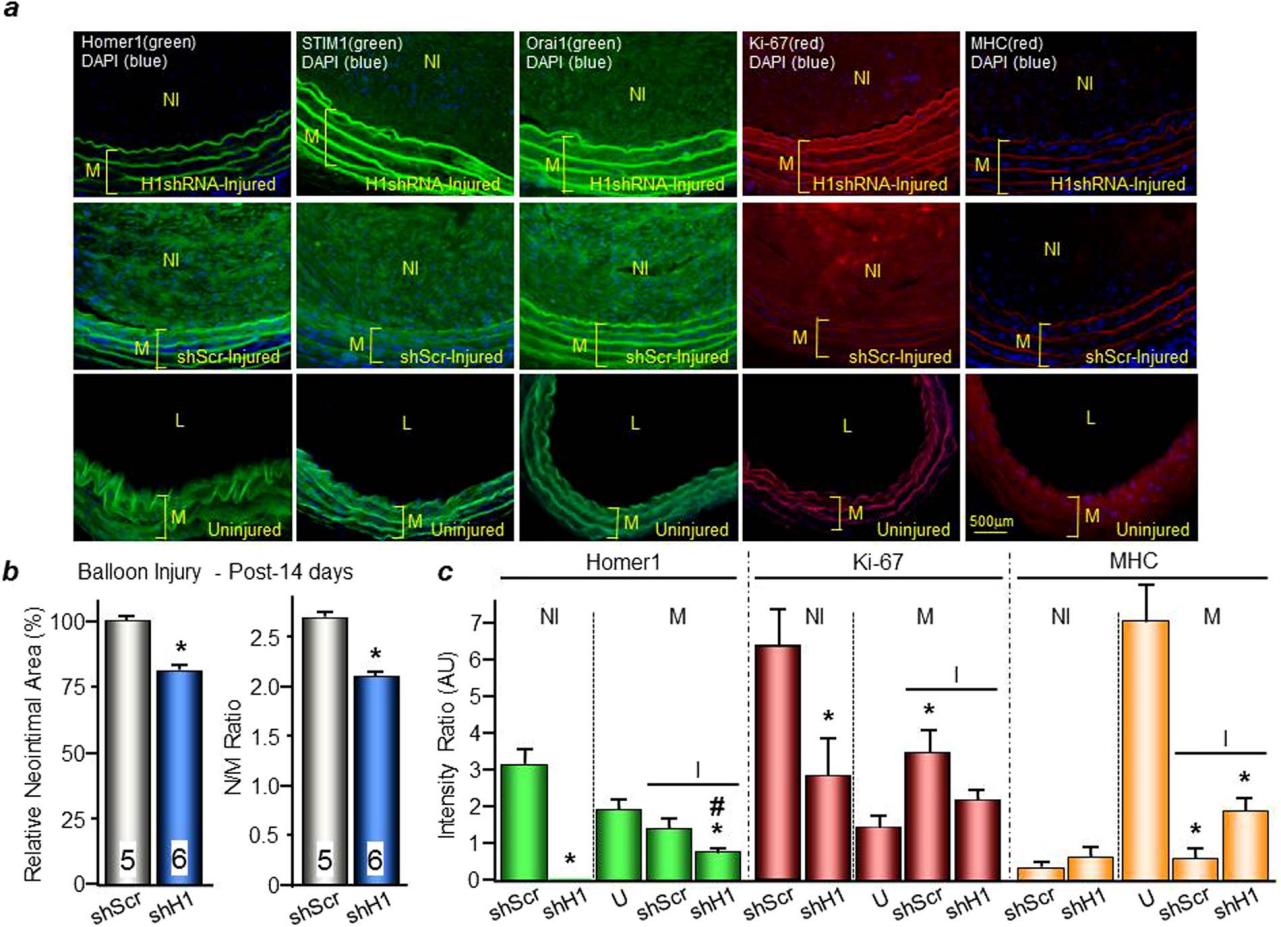
Effect of *in vivo* knockdown of Homer1 on neointima formation. Balloon-injured arteries were treated with AAV-shScrambled(-shScr) or AAV-shHomer1. (**a**) Representative immunofluorescence staining images of Homer1, STIM1, Orai1, Ki-67, and MHC with DAPI in injured (shScr- or shHomer1-treated) and uninjured arteries. (**b**) Quantification of neointimal area in shScr- and shHomer1-treated injured arteries. Neointima area in shScrambled (shScr)-treated arteries is set to 100%. *p < 0.05 or better compared to shScr. (**c**) Fluorescence Intensity Ratios of Homer1, Ki-67, and MHC. Intensities are normalized to DAPI (DNA fluorescence); AU, arbitrary units. *p < 0.05 or better compared to shScr or uninjured arteries in corresponding category (NI or M) and stain; ^#^p < 0.05 compared to shScr in media. n = 5 for shScr-treated injured arteries; n = 6 for uninjured and shHomer1-treated injured arteries. NI = Neointima; M = Media; U = Uninjured; I = Injured.

We extended the findings in cultured VSMCs to the *in vivo* situation by examining the role of Homer in neointima formation. After balloon injury on the left carotid artery, adeno-associated virus (AAV) encoding shScrambled (shScr) or shHomer1 was infused in the injured segment of the common carotid artery for 30 min and then aspirated out. Immunofluorescence staining was done on these injured arteries treated with the respective AAVs as well as on the right intact (uninjured) arteries. To assess the effects of Homer1 knockdown on neointima formation, we analyzed the (1) size of the neointimal area and (2) the immunofluorescence intensities for Homer1, Ki-67, and MHC expression levels normalized to DAPI (DNA fluorescence) stain (intensity ratio). Ki-67 is a marker for cell proliferation, while MHC is a marker for contractile smooth muscle cells. The results are shown in Fig. 5. Homer1, STIM1, and Orai1 are all highly expressed in the neointima of balloon-injured arteries treated with AAV-shScr (Fig. 5a). However, knockdown of Homer1 reduced the size of the neointima, with (a) the relative neointimal area of the AAV-shHomer1-treated injured arteries (n = 6) at 81.4 ± 2.6% vs. 100 ± 2.3% for AAV-shScr-treated injured arteries (n = 5) and (b) the AAV-shHomer1 neointima/media (N/M) ratio at 2.1 ± 0.1 vs. 2.7 ± 0.1 for AAV-shScr-treated injured arteries (Fig. 5b). In shHomer1 injured arteries, the normalized fluorescence intensities (in arbitrary units, AU) of Homer1 and Ki-67 in the neointima were significantly reduced compared to shScr injured arteries (**Homer1 stain**: shHomer1: 0.02 ± 0.01 AU vs. shScr: 3.13 ± 0.49 AU; **Ki-67 stain**: shHomer1: 2.83 ± 0.96 AU vs. shScr: 6.39 ± 1.11 AU) (Fig. 5c). Also, in the media, Homer1 fluorescence intensity significantly decreased in shHomer1 injured arteries (0.76 ± 0.08 AU) vs. uninjured (1.93 ± 0.23 AU) and shScr injured arteries (1.39 ± 0.23 AU), while Ki-67 fluorescence intensity significantly increased in shScr (3.49 ± 0.64 AU) and trended upwards in shHomer1 (2.35 ± 0.18 AU) injured arteries in the media vs. uninjured arteries (1.45 ± 0.34 AU). As expected, MHC fluorescence intensity in the media was significantly reduced in shScr injured arteries (0.58 ± 0.20 AU) vs. uninjured arteries (7.14 ± 1.13 AU), and shHomer1 (1.88 ± 0.38 AU) did not reverse this effect. Thus, AAV-shHomer1 treatment reduces the neointimal area in injured arteries as well as the expression levels of the proliferative marker, Ki-67, in the neointima, and is in agreement with our observation in cultured VSMCs that knockdown of Homer1 suppresses VSMC migration and proliferation leading to neointima formation. Thus, the scaffolding protein Homer1 likely contributes to VSMC remodeling and phenotypic change to proliferative and migratory states, making it an attractive target for therapy to reduce neointimal size and to alleviate arterial occlusion due to acute stenosis.

## Discussion

In summary, this study identifies and establishes the role of Homer1 in vascular smooth muscle cell store-operated Ca^2+^ entry, migration, and proliferation, resulting in neointima formation. The biochemical and functional findings lead to several conclusions: (a) Homer1 expression levels are upregulated in neointimal VSMCs after balloon injury to carotid arteries; (b) Homer1 and STIM1 each interact with TRPC channels and Orai1 in these injured VSMCs; (c) knockdown of Homer1 in cultured VSMCs results in significant reduction of SOCE and in VSMC migration and proliferation; (d) knockdown of both Homer1 and STIM1 in cultured VSMCs results in an additive, significant reduction of SOCE; (e) *in vivo* knockdown of Homer1 in balloon-injured arteries reduces neointimal area and the expression of the cell proliferative marker, Ki-67, arising from VSMC migration and proliferation. Taken together, these data indicate the significance of Homer1 in occlusive arterial disease.

That Homer1 binds to Orai1 in balloon-injured arteries and is required for VSMC SOCE suggests that Homer1 can regulate Orai1 as well as TRPC channel activity in migratory and proliferative VSMCs. Previously, Homer1 has been shown to interact with STIM1 and Orai1 in human platelets, and sequestration of Homer using a proline-rich peptide attenuates thrombin-induced SOCE and platelet aggregation^13^. Thus, upregulation of Homer1 and SOCE appears to be a feature of structural and functional changes in these two cell types. Future studies aimed at mechanisms of Orai and TRPC channel regulation by Homer should reveal the significance of Homer in VSMC SOCE. The additive effect of Homer1 and STIM1 knockdowns suggests that Homer1 can regulate SOCE independent of STIM1. The effects of Homer on SOCE and VSMC migration and proliferation may not be limited through its interactions with TRPC channels, Orai1, and IP_3_ receptors, and we cannot rule out the possibility of Homer interacting with other cellular proteins. However, it is likely that Homer mediates SOCE by forming a complex with the key SOC components: STIM1-Orai1-TRPCs-IP_3_Rs.

We have demonstrated that downregulation of Homer1 acts as a protective mechanism against neointima formation and occlusive arterial disease. In astrocytes and neurons, Homer1 has been shown to relocate ER tubules to junctional sites in proximity to the plasma membrane and to recruit IP_3_ receptors to ER-PM junctions in dendritic spines^31, 32^. Consequently, Homer1 optimizes Ca^2+^ signaling, ER Ca^2+^ store refilling, and glutamate release^33^. In traumatic neuronal injury, downregulation of Homer1 is neuroprotective by suppressing glutamate excitotoxicity resulting from Ca^2+^ overload^34, 35^. A similar protective mechanism and scenario seems to occur in injured arteries when Homer1 is downregulated, with the end result being suppression of VSMC SOCE, migration, and proliferation and reduction in neointima size.

In conclusion, Homer1 downregulation can be considered for future therapeutic treatment of occlusive arterial disease.

## Methods

### Reagents and solutions

All the siRNA used in the present study were obtained from Integrated DNA Technologies. Rat siRNA sequences were used and are listed in Supplementary Table 1. The efficiency of the siRNAs in vascular smooth muscle cells (VSMCs) was determined by RT-PCR using primers listed in Supplementary Table 1. Adeno-associated viruses (AAVs) encoding scrambled or Homer1 shRNA were obtained from Vector Biolabs. The antibodies used were monoclonal anti-STIM1 (BD Biosciences (Cat #610954)), monoclonal anti-Homer1 (Santa Cruz Biotechnology (Cat #SC-17842)), polyclonal anti-TRPC1 (Proteintech Group (Cat #19482-1-AP)), polyclonal anti-β-actin (Cell Signaling Technology (Cat #4967)), monoclonal anti-Ki-67 Clone MIB-5 (Dako (Cat #M724801-8), monoclonal anti-MYH11 (anti-MHC) (Santa Cruz Biotechnology (Cat #SC-6956)), and the following from Alomone: polyclonal anti-TRPC3 (Cat #ACC-016), polyclonal anti-TRPC4 (Cat #ACC-018), polyclonal anti-TRPC6 (Cat #ACC-017), and polyclonal anti-Orai1 (Cat #ACC-062). Anti-STIM1 and anti-Homer1 antibodies were used for co-immunoprecipitation, whereas anti-TRPC1, anti-TRPC3, anti-TRPC4, anti-TRPC6, and anti-Orai1, and anti-β-actin antibodies were used for Western blotting. Anti-STIM1, anti-Homer1, anti-Orai1, anti-Ki-67, and anti-MHC antibodies were used for immunofluorescence staining. Secondary Alexa Fluor^®^ antibodies from ThermoFisher used in this study include Donkey anti-Rabbit IgG (H + L) (488) (Cat #A21206), Donkey anti-Mouse IgG (H + L) (488) (Cat #A21202), Donkey anti-Rabbit IgG (H + L) (568) (Cat #A10042), and Goat anti-Rabbit IgG (H + L) (568) (Cat #A21069).

### Rat Carotid Artery Balloon Injury

All procedures for maintaining the rats and for the balloon injury procedure were performed in accordance with NIH guidelines and regulations and approved by the Institutional Animal Care and Use Committee of the University of North Texas Health Science Center. Male Sprague-Dawley rats (~400–450 g) (Charles River Laboratories) were anesthetized with ketamine (100 mg/kg) via intraperitoneal injection, and balloon angioplasty was carried out as previously described^36^. Briefly, an embolectomy was performed using a 2F Fogarty balloon (Edwards Lifesciences) that is inserted through a small arteriotomy in the external carotid artery and passed into the common carotid artery (CCA). The balloon was inflated at 2 atm pressure, and the catheter was partially withdrawn. The balloon was then deflated, and the catheter was inserted again through the CCA. This balloon injury procedure was done a total of three times. AAV-scrambled or AAV-Homer1 shRNA was infused in the injured segment of the CCA for 30 min and then aspirated out. After recovery from operation and anesthesia, the rats received a postoperative dose (10 mg/kg) of the oral analgesic Rimadyl (carprofen). Sham-operated animals were subjected to a similar procedure but without balloon insertion and injury.

### Hematoxylin and Eosin (H&E) Staining

Rats were anesthetized using 1–3% isoflurane, then perfused with 1X PBS and fixed with 4% paraformaldehyde. Right (uninjured) and left (injured) carotid arteries were dissected out and embedded in paraffin using Leica EG1150 embedder. Arteries were then sectioned (5 μm) on a Leica RM2255 microtome. Sections were air-dried, and H&E staining was performed using standard protocols.

### Immunofluorescence Staining and Morphometric Analysis

Rats were anesthetized using 1–3% isoflurane, then perfused with 1X PBS and fixed with 4% paraformaldehyde. Right (uninjured) and left (injured) carotid arteries were dissected out, fixed with 4% paraformaldehyde, and placed in cryoprotective embedding medium OCT (Tissue-Tek). Arteries were then frozen and sectioned (20 μm) at −20 °C in a Leica CM1950 cryostat. Sections were air-dried, and immunofluorescence staining was performed using standard protocols. Morphometric analysis of each arterial segment was performed using ImageJ software. Neointimal and medial areas were assessed by tracing and measuring the borders of the lumen and internal and external elastic laminas on anti-Ki-67-stained images taken from a 4× objective. Scale was converted from pixels to mm using a 1-mm scale bar, and these numbers (mm) were used to calculate the neointima/media ratio and the relative neointimal area (%). The average neointimal area of balloon-injured arteries treated with AAV-shScr was arbitrarily set to 100%, and the average neointimal area of shHomer1-injured arteries was calculated as a percentage of the area for AAV-shScr-treated injured arteries.

### Harvesting total protein extract and co-immunoprecipitation

The carotid arteries were longitudinally cut, and the media and neointima were harvested after peeling off the adventitia. Tissues were lysed using 500 μl of RIPA buffer: 50 mM Tris-HCL, pH 8 containing 1 mM NaVO3, 10 mM NaPyrophosphate, 50 mM NaF, 150 mM NaCl, 0.2 mM EDTA, 1% Triton X-100, 0.5% Na-deoxycholate, and 0.1% SDS. The extracts were sonicated, and insoluble material was spun down at 30,000 × *g* at 4 °C for 20 min. For the co-immunoprecipitation experiments, anti-STIM1 or anti-Homer1 antibody (1 μg) was added to cell extract (100 μl) and incubated for 2 h at 4 °C. Then, 50 μl of a 1:1 slurry of protein A Sepharose 4B (Sigma) or Protein G Plus Agarose (Pierce-ThermoFisher) beads were added to the antibody-extract mix and incubated for an additional 1 h at 4 °C. Beads were washed three times for 10 min with RIPA buffer, and proteins were released from the beads with 50 μl of SDS-loading buffer. Half of the eluted protein (25 μl) was loaded onto 8% or 10% tris-glycine SDS–PAGE gel under denaturing and reduced conditions. The gel was then transferred onto PVDF membrane for Western blot analysis.

### Isolation and culturing of VSMCs

Rats were euthanized by CO_2_ and cervical dislocation. The descending aorta was dissected out and placed in ice-cold, sterile 1X PBS solution. After fat tissues, blood clots, and endothelium were removed in DMEM followed by 1X PBS wash, the artery was incubated in 0.6 mg/mL papain solution for 30 min at 37 °C, 5% CO_2_, and 100% humidity. The adventitia was stripped away to expose the vascular smooth muscle layer, which was subsequently cut up into pieces and incubated in a collagenase cocktail-DMEM solution containing 1 mg/mL each of collagenase II and H for 45 min at 37 °C, 5% CO_2_, and 100% humidity, with twice homogenization of VSMCs using syringe and 18G-1-1/2 needle in the incubation period. VSMCs were resuspended in 45% DMEM, 45% F12, and 10% FBS supplemented with L-glutamine, and poured through a cell strainer before incubation in 35-mm dish at 37 °C, 5% CO_2_, and 100% humidity.

### siRNA and Homer1 cDNA transfections in cultured VSMCs

The Neon^®^ Transfection System (Invitrogen) was used to transfect siRNA and Homer1 cDNA into VSMCs, according to the supplier’s recommendation. Briefly, VSMCs were grown to 100% confluency in 35-mm dish (equivalent to ~1 × 10^6^ cells). The cells were trypsinized, washed in 1X PBS solution, and resuspended in R buffer to a final volume of 120 μL. One μg of siRNA was mixed in with VSMCs to a final volume of 10 μL. For the Homer1 rescue experiment, 250 ng of Homer1 cDNA was added to the siHomer1-VSMC mix. Using a 10-μL Neon tip, the VSMC-siRNA mix was electroporated at Pulse Voltage: 1475 V; Pulse Width: 20 ms; Pulse Number: 2. The cells were then plated on fibronectin-coated coverslip (Ca^2+^ measurement), 35-mm dish (migration assay), or 96-well plate (proliferation assay), and incubated at 37 °C, 5% CO_2_, and 100% humidity for 72 hours before start of experiment. Passage #’s 8–15 were used for VSMCs in all groups, and results are compared within the same passage #’s.

### [Ca^2+^]_i_ Measurement

VSMCs were loaded with Fura-2 at 5 μm final concentration and incubated for 1 hour at 37 °C, 5% CO_2_. Cells attached to cover glass that formed the bottom of a perfusion chamber were perfused with prewarmed (37 °C) bath solution containing (in mm): 140 mm NaCl, 5 mm KCl, 1 mm MgCl_2_, 10 mm HEPES, 1 mm CaCl_2_, 10 mm glucose, adjusted to 310 mosm, pH 7.4. Chemicals were diluted in standard or Ca^2+^-free bath solution that contained 1 mm EGTA. [Ca^2+^]_*i*_ was measured at the 340- and 380-nm excitation wavelengths. The emitted fluorescence was collected by digital camera (Nikon) at 510 nm and analyzed using the Elements software (Nikon). Results of SOCE from cells treated with siRNA are presented as a percentage of control (scrambled siRNA) 340/380 ratio. Passage #’s 8–15 were used for VSMCs in all groups, and results are compared within the same passage #’s.

### Migration Assay

After transfection with siRNA, VSMCs were cultured for 48 hours to form a monolayer. A 100-μL pipette tip was used to scrape the bottom of the dish, and the wound area was washed with 1X PBS. Cells were incubated at 37 °C, 5% CO_2_ in culture media containing 0.4% FBS with or without 100 ng/mL PDGF-BB (R&D Systems). Brightfield images were captured at 10X magnification (Nikon) at 15 and 24 hours after scratch wound, and analyzed using ImageJ. The total area of migrated VSMCs in the wound was calculated and normalized to control (scrambled siRNA). Results of migration from cells treated with siRNA are presented as a percentage of control. Passage #’s 8–15 were used for VSMCs in all groups, and results are compared within the same passage #’s.

### Proliferation Assay

After transfection with siRNA and plating onto 96-well plate, VSMCs were cultured for 72 hours. The Vybrant^®^ MTT Cell Proliferation Assay Kit (Invitrogen) was used to determine cell number according to the supplier’s recommendation. Briefly, 10 μL of 12 mM MTT were pre-mixed with 100 μL of phenol-red-free DMEM and, together, replaced the growth media. Cells were incubated at 4 hours, 37 °C, 5% CO_2_ so that the MTT can form the insoluble formazan, whose amount is directly proportional to the cell number. The formazan was then solubilized by addition of 100 μL of SDS-HCl solution provided in the kit and incubation for another 4 hours at 37 °C, 5% CO_2_ to form a color whose absorbance is read at 570 nm. Results of the proliferation from cells treated with siRNA are presented as a percentage of control (scrambled siRNA). Passage #’s 8–15 were used for VSMCs in all groups, and results are compared within the same passage #’s.

### Statistics

Results are given as mean + SEM and significance was analyzed by Student’s *t*-test or by one-way analysis of variance (ANOVA).

## Electronic supplementary material

Supplementary Information
