# Supplementary material for: Homer binds to Orai1 and TRPC channels in the neointima and regulates vascular smooth muscle cell migration and proliferation
Source: Sci Rep. 2017 Jul 11;7:5075. doi: 10.1038/s41598-017-04747-w (PMC5506012; doi:10.1038/s41598-017-04747-w)
Supplement: Supplementary file 1 — Supplementary Information [file 41598_2017_4747_MOESM1_ESM.pdf]

Supplementary Figure 1, Jia, et al

Manuscript Title: Homer binds to Orai1 and TRPC channels in the neointima and regulates vascular smooth muscle cell migration and proliferation

Author List: Shuping Jia, Miguel Rodriguez, Arthur G. Williams, Jr., and Joseph P. Yuan

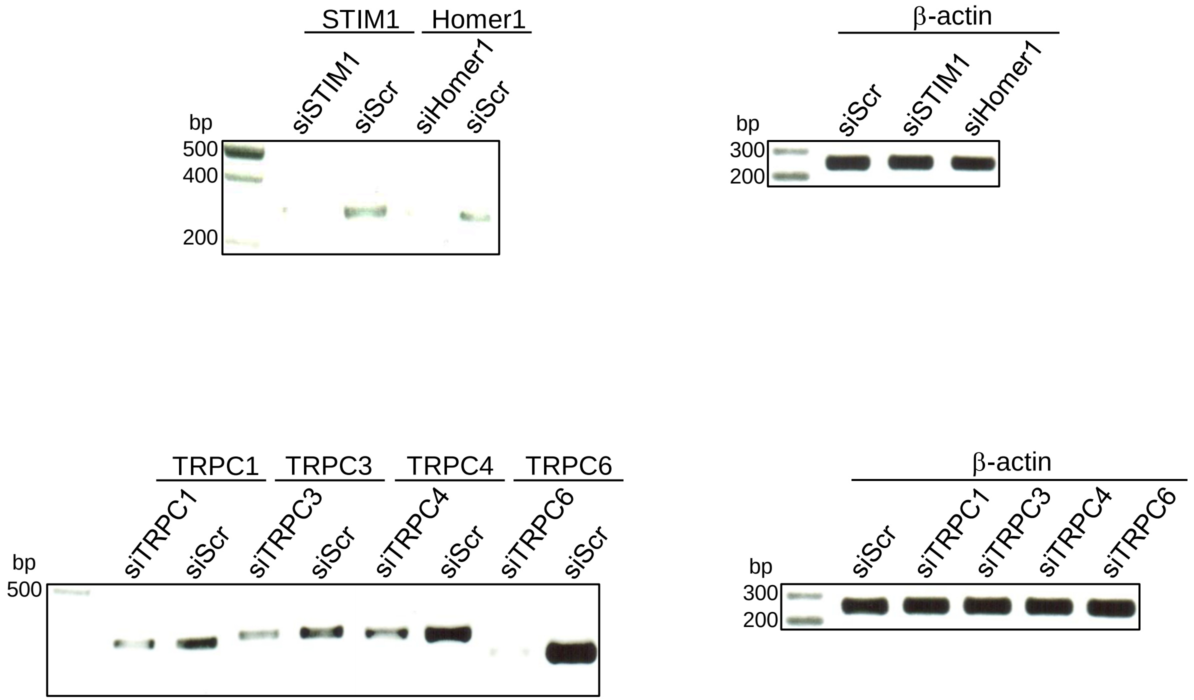

Supplementary Figure 1: **RT-PCR from total mRNA from cultured VSMCs treated with siScrambled, siSTIM1, siHomer1, or siTRPC1/3/4/6**

Complementary DNA (cDNA) expression levels of STIM1, Homer1, and TRPCs are reduced in cells treated with their respective siRNAs. Total mRNA was harvested from cultured VSMCs, and RT-PCR using gene-specific primers was performed according to standard protocols.

Supplementary Figure 2, Jia, et al

Manuscript Title: Homer binds to Orai1 and TRPC channels in the neointima and regulates vascular smooth muscle cell migration and proliferation

Author List: Shuping Jia, Miguel Rodriguez, Arthur G. Williams, Jr., and Joseph P. Yuan

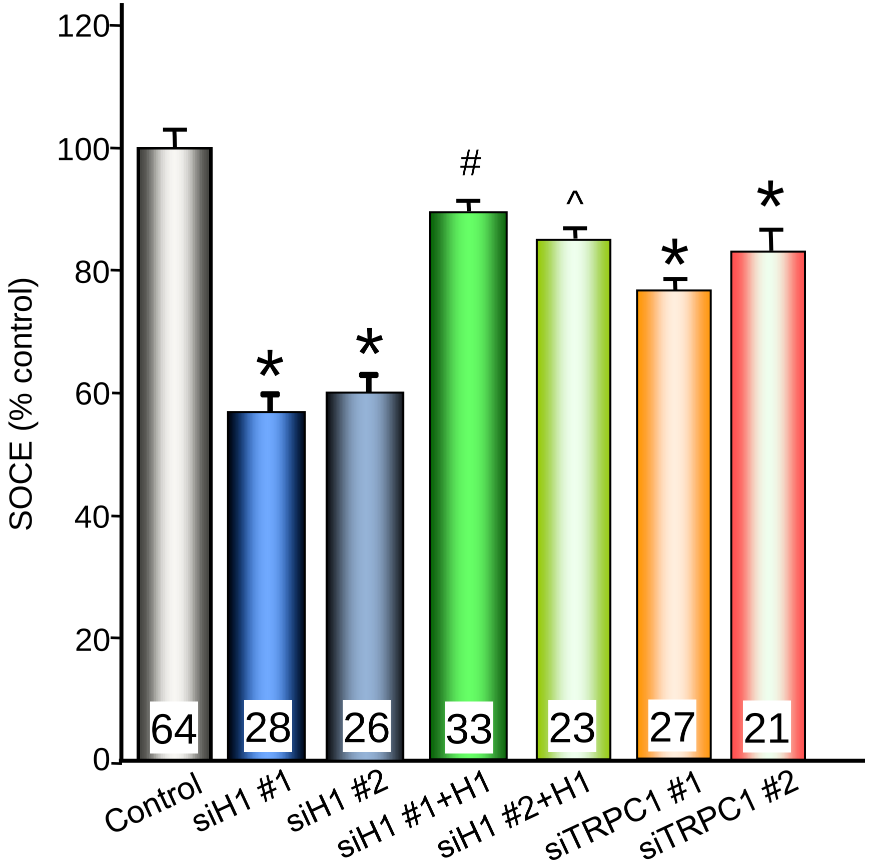

Supplementary Figure 2: **Two independent siRNA's for Homer1 and TRPC1 show similar effects on reducing store-operated Ca^{2+} entry (SOCE) in cultured VSMCs**

Cultured aortic VSMCs were transfected with (**a**) Homer1(H1 #1) and Homer1(H1 #2) siRNAs targeting different regions of Homer1 and (**b**) TRPC1 #1 and TRPC1 #2 siRNAs targeting different regions of TRPC1. SOCE was measured after depleting ER Ca^{2+} stores with 25 mM cyclopiazonic acid (CPA). SOCE values are normalized to peak Ca^{2+} store depletion (CPA response) and indicated as % control (scrambled siRNA), which is set to 100%. SOCE values: siH1 #1: 56.0 ± 4.0%; siH1 #2: 60.0 ± 5.9%; siH1 #1 + H1(250ng): 90.6 ± 3.1%; siH1 #2 + H1(250ng): 88.6 ± 3.3%; siTRPC1 #1: 78.2 ± 2.3%; siTRPC1 #2: 85.6 ± 4.5%. siH1 #1 and siTRPC1 #1 are used in Figures 3 and 4. *, $p < 0.01$ compared to control; $^{\#}$, $p < 0.01$ compared to siH1 #1; ^, $p < 0.01$ compared to siH1 #2.

Supplementary Figure 3, Jia, et al

Manuscript Title: Homer binds to Orai1 and TRPC channels in the neointima and regulates vascular smooth muscle cell migration and proliferation

Author List: Shuping Jia, Miguel Rodriguez, Arthur G. Williams, Jr., and Joseph P. Yuan

Supplementary Table 1: **Sequences for siRNA oligos and list of primers used for RT-PCR**

siRNA oligonucleotides (oligos)

---

siHomer1 #1: 5'-GGAAUUUAGUUAUGAAGAGUUUAAA-3'
siHomer1 #2: 5'-ACCAGAAAGUUAUAAGUAGACACTT-3'
siSTIM1: 5'-GCCAUAGUCACAGUGAGAAGAAUAC-3'
siTRPC1 #1: 5'-GGAUAAAGAAUGGAAGUUUGCUCGA-3'
siTRPC1 #2: 5'-GGAAUACUCAACAACAAUGGAUGTT-3'
siTRPC3: 5'-UGGACAGAAAUGCUAAUUAUGGUCT-3'
siTRPC4: 5'-GGACUCAAGCAUAGAUUAUGAUUTA-3'
siTRPC6: 5'-AGAGUCGAAACAAGAGGAAAGCCGC-3'

RT-PCR primers used to examine mRNA expression in cultured aortic vascular smooth muscle cells (VSMCs)

Homer1 forward primer: 5'-CAAGACAGAGCTGAGTCAGACAGTGCAGGAGC-3'
Homer1 reverse primer: 5'-TCTCTAGGAGTCTTCAGGTTACTGCGGAAAGC-3'
STIM1 forward primer: 5'-CCTGAGGCCCTGCAGAAGTGGCTGC-3'
STIM1 reverse primer: 5'-TATTGACAATCTGGAAACCACAGAGGATCTCGA-3'
TRPC1 forward primer: 5'-AAAGGCAAGGTCAGACGGCAGAACAGCT-3'
TRPC1 reverse primer: 5'-TTGGGATAAAACATAGCATATTTAGAAGTCCGAAAGC-3'
TRPC3 forward primer: 5'-AGGTTAAACCTCTTCACTCAGTCTAACTCGAGAGTTTTTG-3'
TRPC3 reverse primer: 5'-GGTTCAGTTTCTCACTGAGTTTATGAATCAAGATGGC-3'
TRPC4 forward primer: 5'-ATGAGAAGAGCCACAGCGAAGAAGAAATTACTCG-3'
TRPC4 reverse primer: 5'-CAATCTCGTGGTCACATAATCTTCATGGGCA-3'
TRPC6 forward primer: 5'-AACAAACAATCAAGTACAAGGAGCTCAGAAGATTTCC-3'
TRPC6 reverse primer: 5'-TAAAGACAGTCTCTCCCCAAGCTTTCTAATGAGTTCT-3'
β-actin forward primer: 5'-ATGAAACTACATTCAATTCCATCATGAAGTGTGACG-3'
β-actin reverse primer: 5'-CATCGTACTCCTGCTTGCTGATCCACATCTG-3'
